# Protective effects of safranal on kidney tissue in a rat model of distant ischemia-reperfusion injury with infrarenal aortic occlusion

**DOI:** 10.55730/1300-0144.5726

**Published:** 2023-10-12

**Authors:** Mehmet TORT, Hülya SEVİL, Fehim Can SEVİL, Necip BECİT, Uğur AKSU, Zülfükar Kadir SARITAŞ, Hasan Hüseyin DEMİREL, Aziz BÜLBÜL, Zehra YAŞAR, Merve BECİT KIZILKAYA, Hazen SARITAŞ

**Affiliations:** 1 Department of Cardiovascular Surgery, Afyonkarahisar Health Sciences University, Faculty of Medicine, Afyonkarahisar, Turkiye; 2 Department of Emergency, Afyonkarahisar Health Sciences University, Faculty of Medicine, Afyonkarahisar, Turkiye; 3 Department of Cardiology, Afyonkarahisar Health Sciences University, Faculty of Medicine, Afyonkarahisar, Turkiye; 4 Department of Surgery, Afyon Kocatepe University, Faculty of Veterinary Medicine, Afyonkarahisar, Turkiye; 5 Department of Bayat Laborant and Veterinary Health Division, Afyon Kocatepe University, Faculty of Veterinary Medicine, Afyonkarahisar, Turkiye; 6 Department of Physiology, Muğla Sıtkı Koçman University, Faculty of Milas Veterinary Medicine, Mugla, Turkiye; 7 Department of Pharmaceutical Toxicology, Afyonkarahisar Health Sciences University, Faculty of Pharmacy, Afyonkarahisar, Turkiye; 8 Department of Nephrology, Aksaray University, Faculty of Medicine, Aksaray, Turkiye

**Keywords:** Infrarenal aortic occlusion, ischemia and reperfusion, renal injury, safranal

## Abstract

**Background/aim:**

Ischemia-reperfusion (IR) injury to a part of the body can cause damage to distant organs such as the kidney and heart. This study investigated the protective effects of safranal against IR-induced renal injury.

**Materials and methods:**

Used in this study were 24 Wistar Albino male rats, which were divided into 3 equal and randomised groups. The sham group underwent laparotomy only. In the IR group, the infrarenal aorta was clamped for 1 h, and then reperfused for 2 h. In the IR-safranal group, safranal was administered 30 min before the procedure and IR injury was induced in the same way as in the IR group. After the procedure, blood and tissue samples were collected from the rats for biochemical and histopathological analyses. Antioxidant capacity and proinflammatory cytokine analyses were performed on the blood samples. Terminal deoxynucleotidyl transferase-mediated dUTP nick end labeling (TUNEL) staining was performed to determine the number of cells undergoing apoptosis in the kidney tissue.

**Results:**

The estimated glomerular filtration rate, an indicator of renal function, was lower in the IR group (p1 = 0.024 vs. p3 = 0.041, respectively) compared to the other groups, while creatinine levels were higher in the IR group compared to the other groups (p1 = 0.032 vs. p2 = 0.044, respectively). The blood urea nitrogen level was higher in the IR group than in the other groups (p1 = 0.001vs p2 = 0.035, respectively). The total antioxidant and total oxidant status, indicating tissue oxidative stress, did not differ between groups (p = 0.914 vs. p = 0.184, respectively). Among the proinflammatory cytokines, the interleukin-1β (IL-1β) and IL-6 levels were significantly higher in the IR group (p = 0.034 vs. p = 0.001, respectively), but the tumour necrosis factor-α (p = 0.19), and interferon-γ (p = 0.311) levels did not differ between groups. Histopathological examination showed significantly less damage to glomerular and tubular cells in the IR-safranal group (p < 0.001). The number of TUNEL-positive cells was higher in the IR group compared to the other groups (p < 0.001).

**Conclusion:**

Safranal may have protective effects against kidney damage caused by distant ischemia-reperfusion injury.

## 1. Introduction

Ischemia-reperfusion (IR), which may occur during vascular disease and surgery, plays a role in the development of renal failure due to hemodynamic changes, tubular epithelial cell damage, inflammation, and increased tissue oxidative stress [1]. Although numerous methods have been developed to preserve patient renal function and prevent tissue damage, ranging from hemodynamic stabilisation to renal revascularisation, a clear consensus has not yet been achieved in infrarenal aortic disease [2]. Many studies have focused on new treatments to prevent inflammation, prevent renal tissue damage, and preserve renal function [3]. Safranal reduces oxidative stress through its biological effect and prevents apoptosis by suppressing the inflammatory response [4]. The neuroprotective and antiischemic properties of safranal have been demonstrated in many studies [5,6]. The aim of this study was to determine the protective effects of safranal on distant renal injury that may result from IR after infrarenal aortic occlusion.

## 2. Materials and methods

### 2.1. Animals

Used in this study were 24 male Wistar albino rats, weighing 250–300 g (mean 267 g). The laboratory in which they were housed was maintained at 24–26 °C and 55%–60% humidity in accordance with the National Health and Medical Research Council Principles and Guidelines for Experimental Animals and the National Institutes of Health’s Guide for the Care and Use of Laboratory Animals (NIH issue no. 85-23, 1985 revised). The rats were maintained under a 12:12 h light/dark photocycle. They were provided food and water ad libitum until the study procedure began. The study protocol and experimental methods were approved by the Afyon Kocatepe University Animal Experiments Local Ethics Committee (date: 05/06/2020-no:49533702/259).

### 2.2. Study design

The rats were randomly divided into 3 equal groups:

#### Sham group

The infrarenal aorta was dissected by laparotomy but not clamped. After laparotomy, the abdomen was covered with a moist sterile drape and left that way for 3 h. Then, blood and tissue samples were taken.

#### IR group

After laparotomy, the infrarenal aorta was dissected. To achieve ischemia, the infrarenal aorta was clamped and left clamped for 1 h. After removing the clamp to allow reperfusion, blood flow was established after waiting for 2 h.

#### IR-safranal group

According to with the literature, 1 mg/kg safranal was administered intraperitoneally 30 min before laparotomy [7–10]. Then, the same procedures were performed as in the IR group to induce ischemia and reperfusion.

### 2.3. Anesthesia and surgical procedure

Xylazine HCl (Rompun; 23.32 mg/mL 50 mL Viel Bayer, İstanbul, Türkiye) was injected intramuscularly at a dose of 13 mg/kg as premedication, and the rats were anesthetised with ketamine HCl (Ketalar; 50 mg/mL, 10 mL Viel Parke-Davis, Eczacıbaşı, Türkiye) at a dose of 87 mg/kg injected intramuscularly [3,10,11]. In contrast to the other groups, rats in the IR-safranal group received 1 mg/kg of safranal (Sigma-Aldrich: W338907, Merck KGaA, Darmstadt, Germany) 30 min before laparotomy [10,11]. After anaesthesia, the rats were placed on the operating table. The abdominal area was swabbed with antiseptic solution and covered with sterile drapes. After laparotomy from the medial line, the intestinal structures were wrapped with a warm blanket and diverted to the right. The retroperitoneal cavity was entered through a longitudinal incision over the aorta, and the infrarenal aorta was rotated and suspended with elastic bands 1 cm distal to the level of the renal artery and 1 cm proximal to the iliac bifurcation. Anticoagulation was achieved with 150 U/kg intravenous heparin (Koparin, Koçak farma, Türkiye). At 5 min after heparin administration, the infrarenal aorta was occluded with a microvascular clamp. After 1 h of ischemia, the aortic microvascular clamp was removed and 1 mg/kg intravenous protamine sulfate (Promin, Vem, Türkiye) was administered for heparin neutralisation. The peritoneal cavity was closed with sterile drapes. At the end of the second hour of reperfusion, the rats were euthanized with an intraperitoneal injection of 150 mg/kg of thiopental sodium (Pental Na, İ.E. Ulagay, Türkiye) [3,11].

### 2.4. Kidney tissue and blood samples

A 2-mL blood sample was obtained by puncturing the inferior vena cava of each rat before euthanization. The samples were centrifuged and stored at −70 °C for heamatological and biochemical analyses. To assess the histopathological kidney damage and tissue oxidative stress, the right kidneys of the rats were removed.

### 2.5. Biochemical analysis

Hemoglobin, platelet, white blood cell (WBC), and hematocrit counts were obtained from the collected blood samples using a Sysmex XN-2000 hematology system (Sysmex Corp., Norderstedt, Germany). The estimated glomerular filtration rate (eGFR), calcium, creatinine, potassium, sodium, urea, and blood urea nitrogen (BUN) levels were measured from the same blood samples using a Cobas 8000 autoanalyzer (Roche Diagnostics, Basel, Basel-Stadt, Switzerland). The kidney tissue interleukin-1β (IL-1β), IL-6, tumour necrosis factor-α (TNF-α), and interferon-γ (IF-γ) (E-Bioscience, Vienna, Austria) levels were measured using commercial ELISA rat kits with an MVGt Lamda scan 200 (Bio-Tek Instruments, Winooski, VT, USA).

### 2.6. Measurement of the total antioxidant status (TAS) and total oxidant status (TOS)

The TAS and TOS were measured using automated colorimetric methods. TAS values were expressed as mmol Trolox equivalent per gram of protein (mmol Trolox Eq/g protein). TOS values were calibrated with hydrogen peroxide and expressed as micromolar hydrogen peroxide equivalents per gram of protein (μmol H_2_O_2_ Eq/g protein).

### 2.7. Histopathologic evaluation

The tissue samples taken from the rats were fixed in 10% buffered neutral formaldehyde solution for 48 h. They were then embedded in paraffin blocks and cut into 5-μm-thick sections. These sections were stained with hematoxylin and eosin (H&E) and examined by light microscopy. A total of 10 areas were randomly selected from the renal cortical regions and examined at 200X magnification. Lesions observed on the stained slides, including congestion, cellular vacuolization, interstitial oedema, luminal debris, and intratubular detachment, were scored between 0 and 4 points. On observation of no abnormality was scored as 0, mild lesions affecting 25% of the kidney specimens was scored as 1, lesions affecting 25%–50% was scored as 2, lesions affecting 50%–75% was scored as 3, and lesions affecting more than 75% was scored as 4 points [11]. The scores were summed, and the total histopathological damage score was calculated for each group.

### 2.8. Terminal deoxynucleotidyl transferase-mediated dUTP nick end labeling (TUNEL) assay

The In Situ Cell Death Detection Kit, POD, (Cat. No. 1684 817; Roche Diagnostics) was used according to the manufacturer’s instructions. The TUNEL method was performed on tissue sections taken from the 3-aminopropyltriethoxysilane-coated glass slides. The sections were deparaffinized in xylol and rehydrated through a graded series of ethanol. Tissue samples were incubated with proteinase K (pH 8.0, 10 U/mL) for 10 min to detect DNA strand breaks, which were masked by formaldehyde. The sections were rinsed with phosphate-buffered saline (PBS) and then incubated in TUNEL reaction mixture containing fluorochrome at 37 °C for 1 h and rinsed again with PBS. The sections were transferred to a dark environment, a drop of PBS was added, and coverslips were applied. The stained slides were examined under a Zeiss Imager A2 microscope (Carl Zeiss NTS GmbH, Oberkochen, Germany) with a fluorescence attachment. A total of 10 different cortical areas were selected from each kidney and TUNEL-positive cells were counted in those areas. Cells in randomly selected areas of the sections were counted at 40X magnification. Apoptotic indices were obtained by dividing the number of apoptotic cells by the total number of cells (apoptotic index: apoptotic renal tubular epithelial cells in the field / total renal tubular epithelial cells in the field).

### 2.9. Statistical analysis

Data were analyzed using IBM SPSS Statistics for Windows 22.0 (IBM Corp., Armonk, NY, USA). The distribution of the continuous variables was examined using the Shapiro–Wilk test. Results for the descriptive statistics were expressed as the mean ± standard deviation (SD). Statistical comparisons of the continuous variables between the groups were performed using 1-way analysis of variance (ANOVA) or the Kruskal–Wallis test based on their distribution. The Tukey test was used for post hoc analysis after performing the ANOVA. In cases where the Kruskal–Wallis test showed statistical significance, the Bonferroni-corrected Mann–Whitney U test was used to identify the groups with differences. p < 0.05 was considered statistically significant.

## 3. Results

### 3.1. Biochemical values

The parameters of all 3 groups were analysed. The p-value of the statistical analysis between the sham group and the IR group was given as p1, while p2 indicated the analysis between the sham group and the IR-safranal group, and p3 indicated the analysis between the IR group and the IR-safranal group. Hematological evaluation of the blood taken from the rats showed that the hemoglobin (p1 = 0.416, p2 = 0.318, p3 = 0.691), hematocrit (p1 = 0.423, p2 = 0.336, p3 = 0.573), platelet (p1 = 0.988, p2 = 0.832, p3 = 0.750) and WBC (p = 0.449, p2 = 0.995, p3 = 0.445) values were not different between the groups. When the renal function tests were analysed, the eGFR was significantly lower in the IR group (p1 = 0.024, p3 = 0.041). There was no significant difference in the eGFR between the sham group and IR-safranal group (p2 = 0.586). The creatinine level, another parameter of renal function, was higher in the IR group than in the other groups (p1 = 0.032, p3 = 0.044). Similarly, the BUN levels were significantly higher in the IR group than in the other groups (p1 = 0.001, p3 = 0.035). There was no significant difference between the sham group and the IR-safranal group for the either creatinine or the BUN levels (p2 = 0.881 and p2 = 0.094, respectively). The electrolyte evaluation showed that the calcium levels did not differ between groups (p1 = 0.853, p2 = 0.258, p3 = 0.605). Potassium levels were higher in the IR group (p1 = 0.025, p3 = 0.048), and sodium levels were lower in the IR group than in the other groups (p1 = 0.009, p3 = 0.047). There was no difference in the potassium and sodium electrolyte levels between the sham group and the IR-safranal group (p2 = 0.204 and p2 = 0.375, respectively) (Table 1).

Colorimetric measurements showed that the TAS and TOS values were higher in the IR group, but the difference between the groups was not statistically significant (p1 = 0.914, p2 = 0.891, p3 = 0.626 vs. p1 = 0.184, p2 = 0.468, p3 = 0.772, respectively). In the analysis of the proinflammatory cytokines, the levels of all cytokines were high in the IR group. However, this increase was only statistically significant for IL-1β (p1 = 0.034, p2 = 0.126, p3 = 0.086) and IL-6 (p1 = 0.001, p2 = 0.174, p3 = 0.036). This increase was not statistically significant for the other cytokines, such as TNF-a (p1 = 0.19, p2 = 0.216, p3 = 0.423) and IF-γ (p1 = 0.311, p2 = 0.104, p3 = 0.073) (Table 2).

### 3.2. Histopathology

#### 3.2.1. Hematoxylin and eosin (H&E) staining

**Figure 1:**
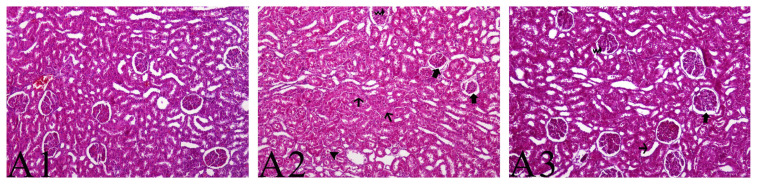
Histopathological evaluation. Hematoxylin-eosin (H&E) staining, A1: sham group, A2: IR group, A3: IR-safranal group (A2 and A3: thick arrow: enlargement of the Bowman’s space in the glomeruli, thin arrow: degenerative and necrotic changes in the tubular epithelial cells, curled arrow: vacuolization formations in the glomerular capillaries, okbase: hyaline cylinder formations in the tubular lumens).

Examination of the glomerular and tubular epithelial cells showed that all of these structures were normal in the sham group. Evaluation of the Bowman’s space enlargement in the glomerulus, degenerative and necrotic changes in the tubular epithelial cells, vacuolization in the glomerular capillary bundle, and inflammatory cell infiltration in the interstitial area showed an increased rate of tissue damage in the IR group. The damage was significantly higher in the IR group compared to the IR-safranal group (p < 0.001) (Table 3). Damage and tubular changes in the glomeruli are shown in Figure 1.

#### 3.2.2. TUNEL assay

**Figure 2:**
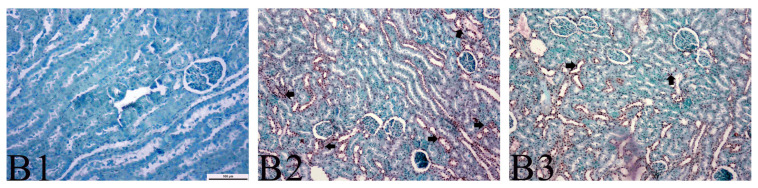
TUNNEL staining. B1: Sham group, B2: IR group, B3: IR-safranal group (B2 and B3: TUNEL-positive cells are indicated by bold arrows).

The number of TUNEL-positive cells was higher in the IR group than in the other groups (p < 0.001) (Table 3). As shown in Figure 2, the TUNEL staining revealed more TUNEL-positive cells in the IR group than in the IR-safranal group.

## 4. Discussion

Abdominal aortic clamping is a commonly used tecnique, particularly in abdominal aortic surgery. Revascularisation of a large ischemic area by occlusion or clamping of the infrarenal aorta can lead to a number of systemic problems characterised by metabolic acidosis, hyperkalemia, and myoglobinemia [12]. This can lead to damage to distant organs such as the heart and kidneys [3]. Despite advances in open surgery, endovascular techniques and intensive care follow-up, complications that can develop after revascularisation of the ischemic limb remain an important cause of morbidity and mortality [13,14]. Renal failure, one of these complications, occurs in 15%–22% of patients after infrarenal aortic surgery and leads to permanent renal failure in some patients [15,16]. The inflammatory response induced by reperfusion following ischemia also plays an active role in renal failure [3,17]. Saffron is a plant grown in Eastern, Middle Eastern, and some European countries and its constituents have potent antioxidant and antiinflammatory properties [18]. Saffron has 4 active metabolites: crocin, crocetin, safranal, and picrocrocin. Many studies on antiinflammatory and antioxidant properties are based on crocin metabolism [19]. However, few studies have been conducted on the antiinflammatory and antioxidant effects of safranal. Most of these studies have investigated antioxidant effects on brain tissue [20]. The fact that the current study was conducted with safranal and not with crocin, one of the active metabolites of bile, is one of the main differences that distinguished this study from others. In addition, the effect of safranal, the subject of the study, on distant organ damage caused by IR is one of the first studies in this field. This study investigated the protective effect of safranal on renal function and tissue in an infrarenal aortic occlusion-induced reperfusion model.

A decrease in the eGFR, one of the renal function tests, is associated with increased mortality and morbidity. In the current study, the eGFR was significantly lower in the IR group than in the other groups, but there was no difference between the IR-safranal group and the sham group. The creatinine level, another parameter of renal function, was significantly higher in the IR group than in the other groups, but as with the eGFR, there was no difference between the IR-safranal group and the sham group. The BUN level is one of the simplest and most commonly used methods to assess renal function [21]. Kidney damage and consequent deterioration in renal function leads to an increase in the BUN level. In the present study, the BUN level was higher in the IR group than in the other groups. On the other hand, there was no statistical difference between the IR-safranal group and the sham group. Hyperkalemia can be seen indirectly in acute kidney injury and acute renal failure [22]. In the current study, the potassium levels were statistically significantly higher in the IR group compared with the other groups. There was no statistical difference in the potassium levels between the IR-safranal and sham groups. In light of these data, it can be said that safranal has a protective effect on renal function.

After IR, inflammatory cytokines and adhesion molecules induce oxidative stress in the tissue. It has been reported in the literature that TAS and TOS levels increase with IR injury [3,23]. In the present study, oxidative stress was assessed by measuring the TAS and TOS levels in the tissue. The TAS and TOS levels were higher in the IR group, but this increase was not statistically significant when compared to the other groups. In an experimental study by Collino et al. [24], it was reported that the IL-1β, IL-6, and TNF-α levels were significantly increased in the IR group compared to the sham group after IR. Proinflammatory cytokines IL-1β, IL-6, TNF-α, and IF-γ, which are released after IR and contribute to tissue damage, trigger many cascades of events [25,26]. In the present study, all of the proinflammatory cytokine levels were high in the IR group, but the IL-1β and IL-6 levels were statistically significantly higher. Although there was no statistically significant difference in the cytokine levels, other than IL-1β and IL-6, these levels were lower in the IR-safranal group than in the IR group. When the effect of safranal on the proinflammatory cytokines was evaluated in comparison with other studies [27], it is our belief that the lack of statistical significance between groups in regard to the proinflammatory cytokines, other than IL-1β and IL-6, in the current study may have resulted based on the dose of safranal used.

Glomerular and tubular damage leading to renal failure is an event that occurs after IR and increases morbidity and mortality [17]. The current study has shown that safranal, which has beneficial effects on renal function, also provides structural protection to renal tissue. It was shown herein that safranal reduced the enlargement of the Bowman’s space in the glomerulus and reduced degenerative and necrotic changes in the tubular cells. In addition, safranal was found to significantly prevent the formation of glomerular vacuolation and the infiltration of inflammatory cells into the interstitial space.

The effect of IR injury on cells is complex. It is known that calcium overload caused by ion channel imbalance, excessive reactive oxygen species production, inflammatory response, and protein kinase activation cause damage to mitochondria and endoplasmic reticulum, leading to apotosis and necrosis in these cells [28]. The TUNEL test detects DNA breaks. Since DNA breaks occur at all stages of apoptosis, TUNEL staining is used as an indicator of apoptotic cell death [29]. In the current study, TUNEL staining was used to determine the extent of the cell apoptosis. The apoptotic index calculated from the TUNEL assay test showed that IR caused an increase in the level of tissue apoptosis, whereas safranal decreased the level of tissue apoptosis. All of these histopathological evaluations support the finding that safranal has a protective effect on kidney tissue.

### 4.1. Limitations

The IR experimental model in the current study was established by collecting samples from the rats after 1 h of ischemia and 2 h of reperfusion, in accordance with previous studies [3,10,11]. In addition, safranaline was administered as a single dose of 1 mg/kg [10,30]. However, it is known that the effect of an agent on the living system depends on the dose, route, and duration of administration. Depending on these factors, its efficacy may be reduced or increased. In the present study, there was no clear consensus on the effective dose [7,30], duration, or route of administration of the safranal, and no studies on remote organ damage caused by IR injury could be found in the literature. This was the main limiting factor of the study. Although this study provided significant data that safranal may be beneficial for distant organ damage due to IR, randomised controlled trials with more subjects are needed to determine the effective dose and duration of administration.

In addition to these factors, because this study was conducted on rats weighing 250–300 g, it was not possible to obtain sufficient urine samples from each of them. Taking into account the factors of equality between groups and minimum data requirements, the results of the urine samples were not included in the study to avoid possible statistical errors.

## 5. Conclusion

Based on the data from this study, it is our belief that safranal may have protective effects against kidney damage caused by distant IR injury.

## Figures and Tables

**Table 1:** Serum hematological and biochemical levels of the groups (n = 8) (mean ± SD).

	Sham group	IR group	IR-safranal group	p1	p2	p3
Creatinine (mL/dL)	0.40 ± 0.11	0.53 ± 0.10	0.43 ± 0.03	**0.032**	0.881	**0.044**
eGFR (mL/min/1.73 m^2^)	129.12 ± 9.58	117.80 ± 7.52	125.50 ± 3.29	**0.024**	0.586	**0.041**
BUN (mg/dL)	21.00 ± 1.64	35.28 ± 4.93	32.86 ± 4.00	**0.001**	0.094	**0.035**
Hemoglobin (g/dL)	15.01 ± 1.14	15.81 ± 0.91	15.23 ± 0.47	0.416	0.318	0.691
Platelet count (10^9^/L)	443 ± 179.90	432.62 ± 144.34	483.75 ± 77.53	0.988	0.832	0.75
WBC (10^9^/L)	4.21 ± 1.57	3.09 ± 1.57	4.21 ± 2.19	0.449	0.995	0.445
Hematocrit (%)	40.77 ± 3.40	40.13 ± 2.73	40.62 ± 1.40	0.423	0.336	0.573
Calcium (mg/dL)	10.82 ± 1.29	10.51 ± 0.81	9.96 ± 0.94	0.853	0.258	0.605
Potassium (mmol/L)	4.60 ± 0.87	5.79 ± 0.58	5.35 ± 0.91	**0.025**	0.204	**0.048**
Sodium (mmol/L)	139.37 ± 4.37	131.80 ± 4.65	134.87 ± 2.85	**0.009**	0.375	**0.047**

BUN: blood urea nitrogen, eGFR: estimated glomerular filtration rate, WBC: white blood cell. p1: Statistical analysis between the sham group and IR group, p2: statistical analysis between the sham group and IR-safranal group, P3: statistical analysis between the IR group and IR-safranal group. Data are presented as the mean ± SD. Bold p-values are statistically significant among the groups (p < 0.05).

**Table 2:** Inflammatory marker and oxidative parameter levels in the kidney tissues of the groups (n = 8) (mean ± SD).

	Sham group	IR group	IR-safranal group	p1	p2	p3
TAS (U/m L)	4.03 ± 0.63	4.19 ± 0.47	3.90 ± 0.75	0.914	0.891	0.626
TOS (U/m L)	4.82 ± 0,78	5.62 ± 0,96	5.34 ± 0,64	0.184	0.468	0.772
IL-1β (ng/L)	4.68 ± 0,83	5.71 ± 0,85	5.46 ± 0,32	**0.034**	0.126	0.086
IL-6 (ng/L)	2.80 ± 0.65	3.94 ± 0.31	3.49 ± 0.46	**0.001**	0.174	**0.036**
TNF-α	8.30 ± 1.27	9.65 ± 1.39	9.46 ± 1.43	0.19	0.216	0.423
IF-γ (ng/L)	12.34 ± 2.54	13.15 ±2.05	10.53 ± 2.15	0.311	0.104	0.073

TAS: Total antioxidant status, TOS: total oxidant status, IL: interleukin, TNF: tumor necrosis factor, IF: interferon. Data are expressed as the mean ± SD. Bold p-values are statistically significant between the groups (p < 0.05).

**Table 3:** Histopathological analysis of the groups.

	Sham group	IR group	IR-safranal group	p1	p2	p3
Enlargement of Bowman’s space in glomeruli	0	2.12 ± 0.59	0.87 ± 0.78	**0.001**	**0.001**	**0.001**
Degenerative changes in tubular epithelial cells	0	1.87 ± 0.33	0.73 ± 0.63	**0.001**	**0.001**	**0.001**
Vacuolization of glomerular capillary lumps	0	2.1 ± 0.51	1.1 ± 0.32	**0.001**	**0.001**	**0.001**
Hyaline cylinder formation in the tubular lumen	0	1.76 ± 0.66	0.73 ± 0.63	**0.001**	**0.001**	**0.001**
Apoptotic index in tunnel staining	5 ± 0.89	41.5 ± 5.12	29.66 ± 3.82	**0.001**	**0.001**	**0.001**

Data are expressed as mean ± SD. Bold p-values are statistically significant between the groups (p < 0.05).
